# Antimicrobial Resistance in the COVID-19 Landscape: Is There an Opportunity for Anti-Infective Antibodies and Antimicrobial Peptides?

**DOI:** 10.3389/fimmu.2022.921483

**Published:** 2022-06-02

**Authors:** José M. Pérez de la Lastra, Uttpal Anand, Sergio González-Acosta, Manuel R. López, Abhijit Dey, Elza Bontempi, Antonio Morales delaNuez

**Affiliations:** ^1^ Biotechnology of Macromolecules, Instituto de Productos Naturales y Agrobiología, IPNA (CSIC), San Cristóbal de la Laguna, Spain; ^2^ CytoGene Research & Development LLP, Barabanki, Uttar Pradesh, India; ^3^ Department of Life Sciences, Presidency University, Kolkata, India; ^4^ National Interuniversity Consortium of Materials Science and Technology (INSTM) and Chemistry for Technologies Laboratory, Department of Mechanical and Industrial Engineering, University of Brescia, Brescia, Italy

**Keywords:** SARS-CoV-2, antibiotic resistance, one health approach, global health, antibiotic discovery, antimicrobial peptides, environmental contamination, vaccination

## Abstract

Although COVID-19 has captured most of the public health attention, antimicrobial resistance (AMR) has not disappeared. To prevent the escape of resistant microorganisms in animals or environmental reservoirs a “one health approach” is desirable. In this context of COVID-19, AMR has probably been affected by the inappropriate or over-use of antibiotics. The increased use of antimicrobials and biocides for disinfection may have enhanced the prevalence of AMR. Antibiotics have been used empirically in patients with COVID-19 to avoid or prevent bacterial coinfection or superinfections. On the other hand, the measures to prevent the transmission of COVID-19 could have reduced the risk of the emergence of multidrug-resistant microorganisms. Since we do not currently have a sterilizing vaccine against SARS-CoV-2, the virus may still multiply in the organism and new mutations may occur. As a consequence, there is a risk of the appearance of new variants. Nature-derived anti-infective agents, such as antibodies and antimicrobial peptides (AMPs), are very promising in the fight against infectious diseases, because they are less likely to develop resistance, even though further investigation is still required.

## Introduction

The COVID-19 pandemic has highlighted the susceptibility of humans to emerging infectious diseases (1). A serious threat to the world’s population is still faced by viral pandemics because many viral diseases have no treatment and because of the emergence or re-emergence of some virus strains. Scientists believe that the SARS-CoV-2 virus was first discovered in animals and then spread to humans by crossing the species barrier. Like all other viruses belonging to the coronavirus family, SARS-CoV-2 can cause infection in both humans and animals, which means that COVID-19 is a zoonotic disease or zoonosis (2, 3). Almost 75% of the emerging pathogens are zoonotic. The emergence of these new resistant microorganisms and their transfer between humans, animals, and ecosystems can be facilitated or impeded because of environmental circumstances and behaviors (4–6). In today’s increasingly globalized society, an infected person is able to spread the disease much faster than hundreds of years earlier. This finding has again highlighted the importance of the one health approach to integrating human health, animal health, and the environment (7).

**Figure 1:**
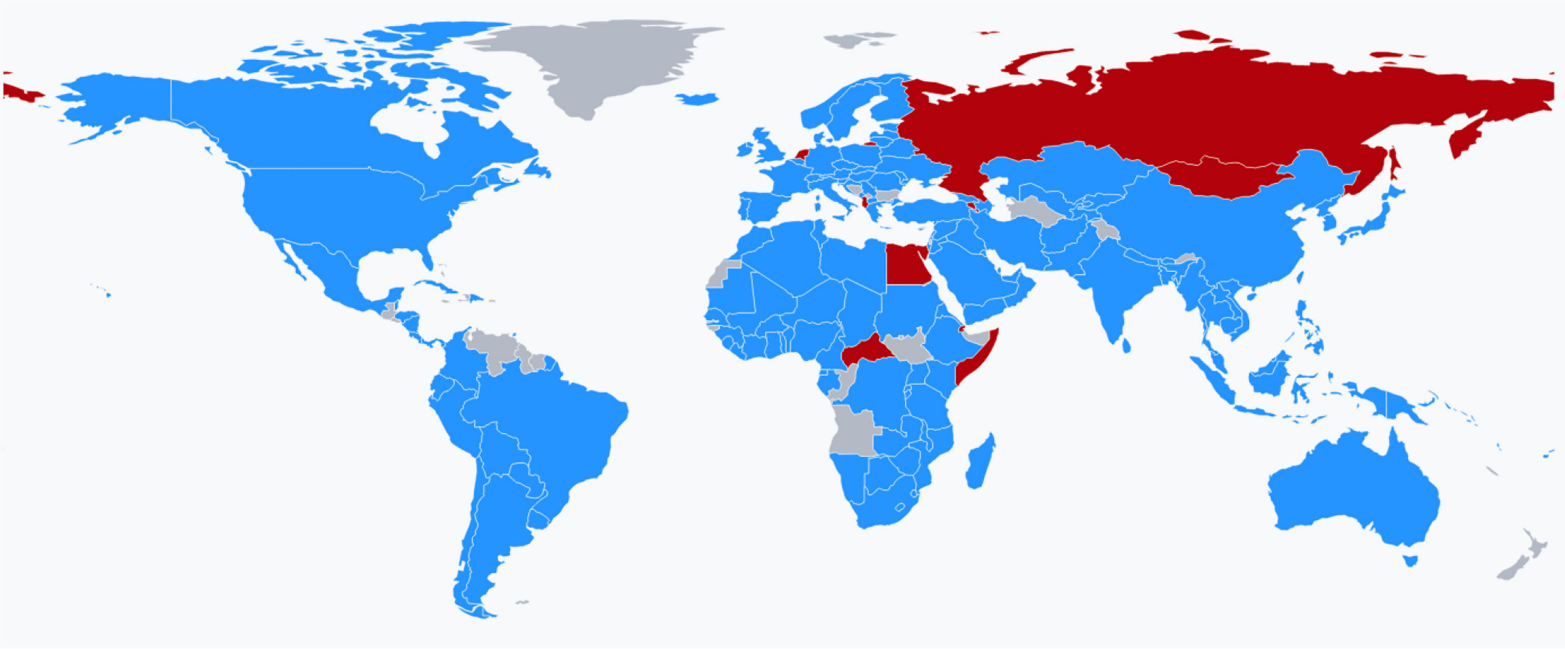
World map showing the countries (in blue) that, during 2020-2021 responded favorably to the question “Has your National AMR Action Plan development and implementation process been affected by the COVID-19 pandemic and the national response in your country?”. Countries in red are those who responded negatively. Source: Global database for the Tripartite Antimicrobial Resistance (AMR) Country Self-Assessment Survey (TrACSS) https://amrcountryprogress.org.

Antimicrobial resistance (AMR) occurs when microorganisms (bacteria, fungi, viruses, and parasites) undergo heritable changes when exposed to antimicrobial agents such as antibiotics, antifungals, or antivirals. The development of resistant strains with a high potential for infection occurs as a result of mutation or re-assortment of pre-existing microbial strains, rendering vaccines and medicines ineffective in some cases (8). The selective pressure exerted by antimicrobials induces mechanisms for the acquisition of resistance in microorganisms, such as spot mutation or horizontal gene transfer, which pass from generation to generation and therefore select microorganisms that have inherited this resistance (9, 10). Although several classes of broad-spectrum antibiotics are available to treat Gram-positive and Gram-negative bacterial infections, many pathogens rapidly evolve or acquire resistance to first-line treatments and respond only to last-resort antibiotics (11, 12). An infection caused by resistant microorganisms is more difficult to treat. Affected people may require hospitalization, generate more clinical complications, and, eventually, may result in the patient becoming a carrier of the AMR with the possibility of transmitting the infection to those around him/her. In most cases, the severity of disease associated with the emergence of resistance depend on the incidence and diversity of infections, as well as the availability, efficacy, and safety of the therapeutic approaches adopted (13, 14). Significant efforts are underway to discover new classes of antibiotics and to develop derivatives and drug combinations (15). The World Health Organization (WHO) warns that by 2050 there will be more deaths from multidrug-resistant bacteria worldwide than from cancer (16). Most countries have revealed that the process and development of their AMR National Action plan has been affected by the COVID-19 pandemic **(** **Figure 1** **)**.

Our goal is to analyze the factors associated with COVID-19 which limit or promote the emergence of AMR. Knowledge of the factors affecting this relationship will help mitigate the impact of the COVID-19 pandemic on AMR. In this scenario, we encourage the investment in research and development of Nature-derived anti-infective agents; such as antimicrobial peptides (17) and antibodies (18), which are distinguished by their limited ability to generate resistances (19).

## Impact of COVID-19 on Antibiotic Prescription

**Figure 2:**
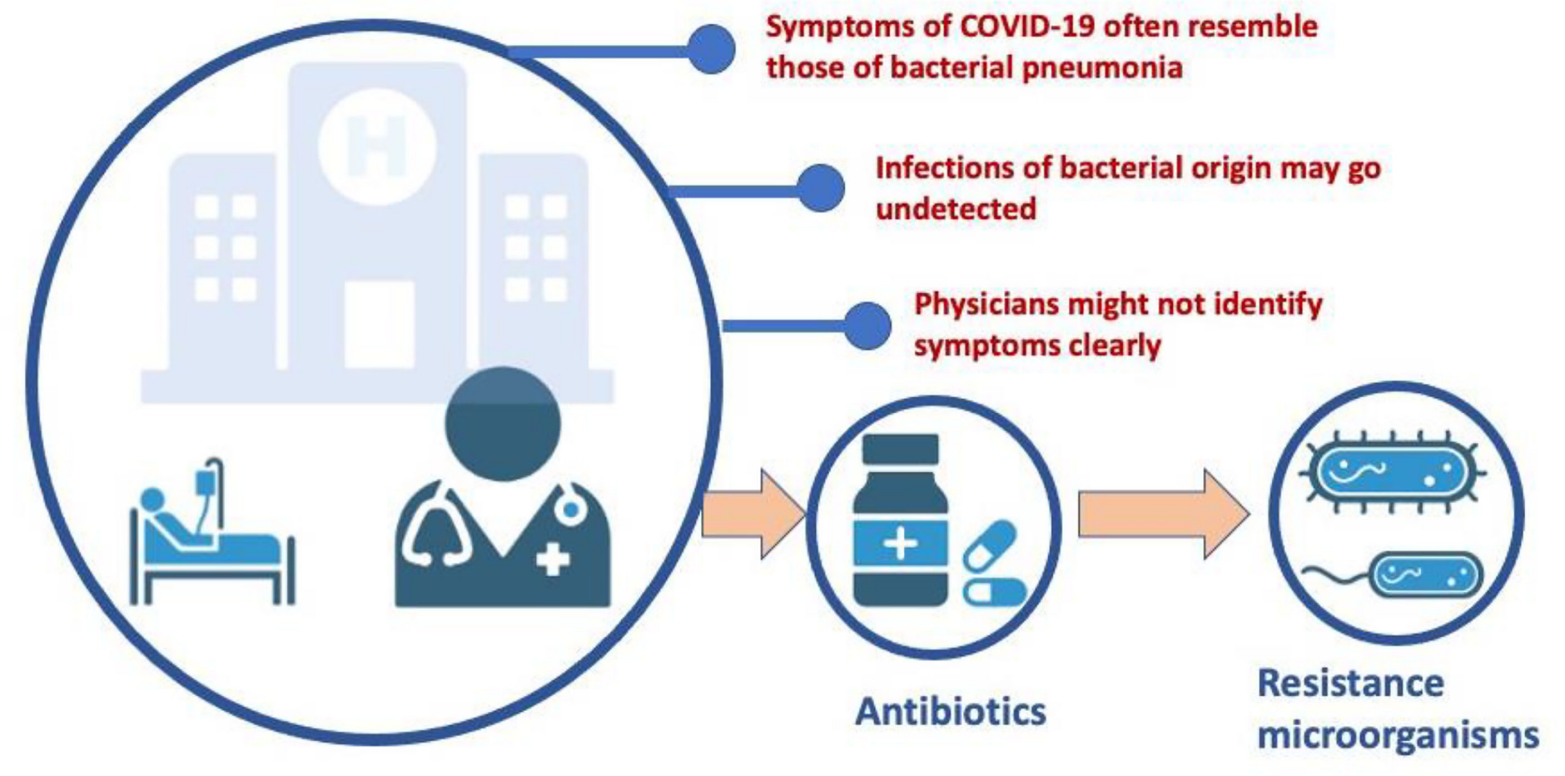
Impact of COVID-19 on antibiotic prescribing. One of the potential consequences of the COVID-19 pandemic is the spread of antimicrobial resistance in the acute care setting as a result of increased antimicrobial use. Although antibiotics cannot destroy viruses, physicians are sometimes insufficiently aware of the symptoms and natural course of respiratory infectious diseases and often prescribe antibiotics to those diagnosed patients in whom there is confirmation or high suspicion of bacterial coinfection or superinfection. Consequently, many hospitalized patients with COVID-19 are prescribed empirical antibiotics, often in the absence of microbiological confirmation of the diagnosis.

At social level, COVID-19 has enabled greater visibility of infectious diseases (1, 20). One potential consequence of the COVID-19 pandemic is the spread of antimicrobial resistance in the acute care setting because of the increased antimicrobial use (21). In hospitals, antibiotics have been used intensively in patients with COVID-19 to eliminate potential bacterial infections (22, 23). Up to 70% of patients with COVID-19 receive antibiotic treatment, either on an outpatient or inpatient basis. Antibiotics cannot destroy viruses. However, physicians often need to prescribe antibiotics to hospitalized patients with COVID-19 who have a confirmed or strong suspicion of bacterial coinfection or superinfection (24). Sometimes physicians do not have sufficiently knowledge of the symptoms and natural course of respiratory infectious diseases and prescribe empiric antibiotics, even if the diagnosis is not microbiologically confirmed. One of the main reasons of antibiotics prescription is due the symptoms of COVID-19, that often resemble to those of bacterial pneumonia (25). Diagnostics used to distinguish viral from bacterial pneumonia may be ineffective or have response times of hours or days, while immediate treatment is needed. In addition, bacterial infections in patients who do not have COVID-19 can go unnoticed and require delayed treatment when all focus is on pandemic control. Patients with COVID-19 may also be affected by a secondary bacterial infection requiring antibiotic treatment, contributing to an increased use of these drugs (26–28) (**Figure 2**). There is a risk that a large number of people may use antibiotics in an erroneous self-medication attempt to protect themselves from the virus. This might be particularly common in underdeveloped countries where antibiotics can be available without a prescription.

Currently, the impact of the pandemic on the prevalence of multidrug-resistant (MDR) bacteria is still unclear. More and better data are needed to better understand the incidence of co-infections and the pathogens involved, as well as the impact of underlying risk factors on patients. Recent reports have described a high use of broad-spectrum antibiotics as a risk factor in the emergence of multidrug-resistant microorganisms, which often appear in critically ill COVID-19 patients (29–31).

## Impact of COVID-19 on the Release of Antimicrobials into the Environment

Habitat degradation is an important factor in the increasing threat posed by pandemics and other human health problems. The destruction of forests and the expansion of urban areas and industrial activity can be dangerous for a wide range of animal species. The survivors are forced to be closer to each other and humans, which makes it more likely that harmless animal microbes will turn into deadly human pathogens. The COVID-19 pandemic is supposed to have begun in bats and spread from a “wet market” in Wuhan, China, where live wildlife species are available for human consumption. Selling wild animals for human use, whether as pets or in the form of live animal markets, plainly poses a significant danger to public health. These marketplaces serve as breeding grounds for zoonotic infections by housing ill, stressed, and overcrowded animals in extremely unclean settings. The continued use of these procedures will cause further disasters to human health in the future, with the potential for much greater devastation.

A key factor in the development of bacterial resistance is the ability of the microorganism to adapt rapidly to new environmental conditions (32). In industrial livestock models, the widespread and indiscriminate use of antibiotics and growth promoters exacerbates the problem by producing pathogenic strains resistant to these drugs. Despite bans in different countries, antibiotics are being used in animal husbandry, not only to treat infections but also to promote weight gain in animals such as cattle, pigs, and poultry (33). The overuse of antimicrobial drugs in farm animals and human medicine has been linked to the emergence of multidrug-resistant microbes (34, 35). About 65 percent of all antibiotics used for human treatment (including tetracyclines and penicillins) are marketed for animal use in the United States. Most of them are administered to entire groups of animals, even if none of them are sick. As a result, antimicrobial drugs become ineffective and infections persist in the organism, increasing the risk of spread to other people or animals (36). Humans can become infected with antibiotic-resistant bacteria through handling or eating raw or undercooked meat, coming into contact with livestock or their excrement, and/or eating food or drinking water (including recreational water) contaminated with animal feces. Antibiotics are one of the most frequently found chemicals in aquatic environments worldwide. This might have serious consequences for the ecology as well as the spread of AMR in the environment. Alternative livestock production techniques are promoted by agroecology by reducing pesticide use and increasing soil fertility in ecological ways.

The pandemic led to some livestock animals being kept on farms longer than usual because of problems with transportation and outbreaks at the slaughterhouse. This may have contributed to increasing animal density on farms, which may have resulted in greater antibiotic administration in animal production. Preventive use of antibiotics in farmed animals was recently prohibited by the European Union. It is expected that other nations follow suit for genuine success in the battle against AMR.

**Figure 3:**
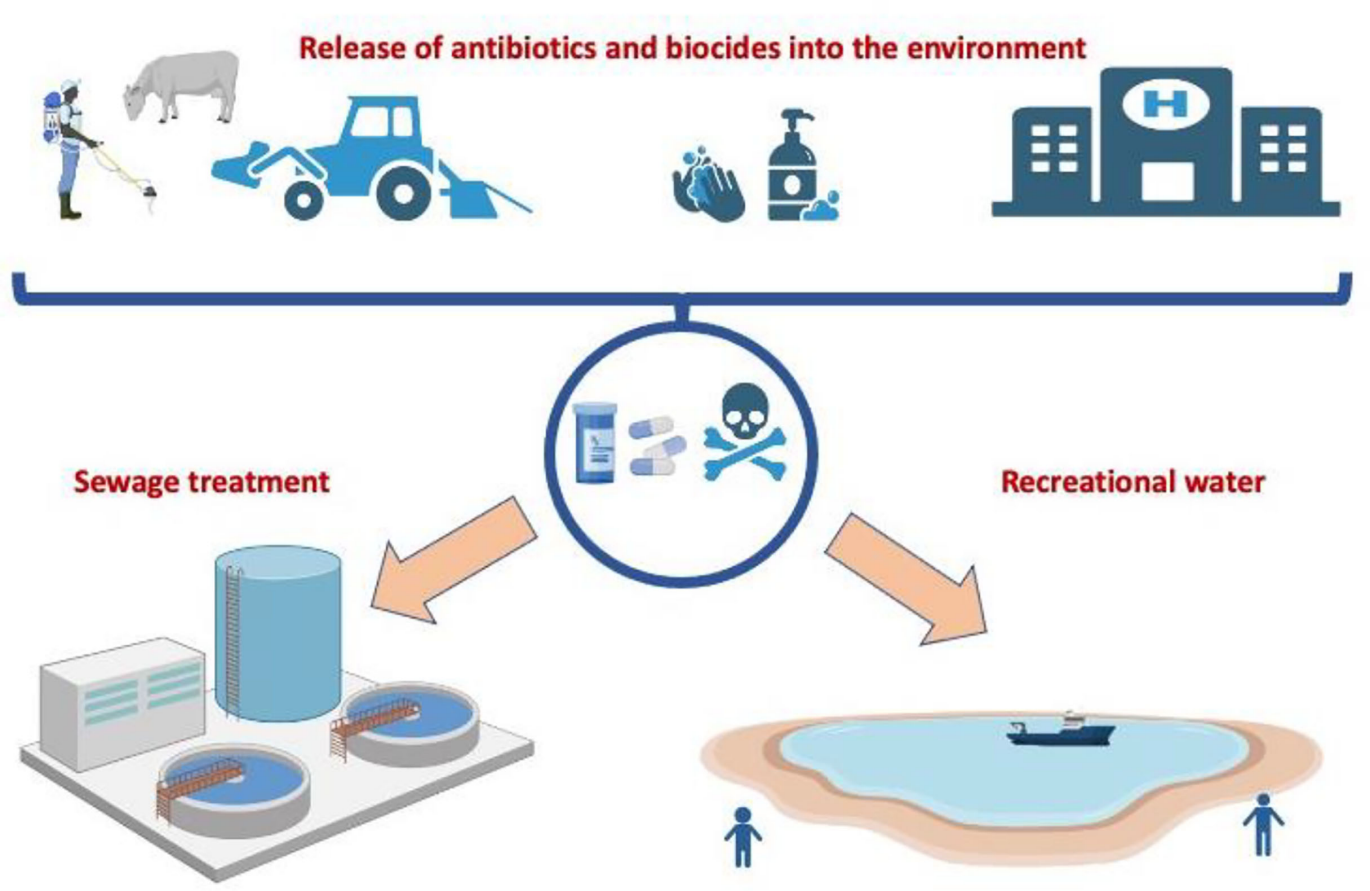
Impact of COVID-19 on the release of antimicrobials into the environment. The excessive use of antimicrobial drugs in farm animals and human medicine, especially in hospitals, can lead to an increase in the concentration of biocides and disinfectants in wastewater and sewage treatment plants and cause the selective survival of bacterial strains carrying resistance genes.

Hand washing is considered an essential means of preventing nosocomial infections, mostly in healthcare settings. Hand hygiene is highly recommended to prevent the acquisition and transmission of SARS-CoV-2 infection (37). The advent of COVID-19 has led to an increase in the consumption of antibacterial soaps, hydroalcoholic gels, and other handwashing products and disinfectants (38, 39). It has become so popular that, at least in developed countries, almost all stores, schools, hospitals, and workplaces carry these hand hygiene products to prevent the acquisition and transmission of infections (38, 40). Hand hygiene products typically contain bactericidal, fungicidal, and virucidal products, as well as alcohol and non-alcohol detergents, but some manufacturers add antimicrobial compounds to make the product more effective against pathogens (41). These chemicals, like hydrogen peroxide and sodium hypochlorite, peroxyacetic acid, and chlorine dioxide may penetrate soil systems and damage native biota. Environmental remediation and biogeochemical cycling of elements might be disrupted by the introduction of these chemicals into soil. The presence of these biocides can interfere with wastewater treatment methods that rely on the activity of microorganisms that play key roles in biogeochemical cycles and environmental remediation (42–44). Although ethanol is the predominant ingredient in most hand hygiene products, some gels also incorporate other synthetic substances that may not offer much in terms of protection but instead may fuel bacterial antimicrobial resistance (45). This may result in the selective survival of bacteria harboring resistance genes, and in the development of AMR (46–48). During the COVID-19 crisis, the massive use of these substances, in particular in hospitals, and the continued use of these hand hygiene products can lead to unintended release of biocides and disinfectants into wastewater and sewage treatment plants (49, 50). As a result of extraordinarily high bacterial loads combined with subtherapeutic drugs, wastewater is a significant source of AMR, causing the selective survival of bacterial strains carrying resistance genes (6, 39) (**Figure 3**).

## Impact of COVID-19 Vaccination on the Emergence of Resistance

The use of vaccines may minimize resistance to the pathogens targeted by vaccination. Vaccines are an important tool in the prevention of infections and have had a positive impact on the reduced use of antibiotics and the development of antibiotic resistance (51). Vaccination prevents infectious diseases and their complications; is easy, cheap, and saves lives (52). Conventional vaccines may lower viral loads, thereby decreasing onward transmission. Vaccine protection is twofold: it protects the inoculated person to prevent infection, and on the other hand, it can protect the surrounding people, even those who are not immune to the disease, since the immunized person is unable to spread the infection, a concept named “herd immunity” (53). When a vaccine stops people from getting the virus or bacteria, it gives them sterilizing immunity. This means that they can’t have enough viruses in their bodies to spread to other people. In contrast, vaccines that cause “non-sterile” immunity protect the host from the disease but do not stop the disease from spreading. prevent the emergence of new variants and reduce the development of resistance, it is essential that the virus cannot replicate among infected people, so vaccines that provide sterilizing immunity or measures that prevent the colonization of the virus in tissues are desirable (54, 55). The administration of COVID-19 vaccination, and other vaccines for simultaneous co-infections, are anticipated as a safe and effective measure to prevent infections (56) and further evolution of variants (57). The new COVID-19 vaccinations have the potential to reduce the usage of antibiotics for COVID-19 patients (58). However, current COVID-19 vaccines do not prevent vaccinated people from infecting themselves and others. As a result, anybody who contracts the virus, regardless of vaccination status, has the potential to spread it, which makes herd immunity an unrealistic goal (59). Given that the scientific community currently accepts that both vaccinated and unvaccinated people can be a source of infection for others, the sanitary utility of the green pass for the prevention of COVID-19 infection is questionable (60, 61). Without additional control measures to discriminate against infection, this action could enable to an increase in the burden of disease among those vaccinated in these local contexts required by the passport.

The rapid development of COVID-19 vaccines and the fear of side-effects has raised doubts about the safety and efficacy of vaccination among certain groups of people (62). If the benefits/risks of vaccination are not correctly explained to the population, this perception could negatively affect the acceptance of “normal” vaccines, such as measles and polio. Focusing excessive attention on the pandemic could also have led to substantial disruption of other global health programs, including routine childhood vaccination campaigns against cholera, measles, meningitis, polio, tetanus, typhoid, and yellow fever. This situation could increase the number of people with no defense against these diseases and their associated complications, and the occurrence of resistance (63, 64).

As long as the virus continues to be spread, there is the possibility that new variants may appear that are more contagious, produce more severe symptoms, or evade the effect of vaccines (65, 66). The rapid global spread of the delta variant was explained by the maintained viral load despite increasing vaccination coverage (67, 68). Current vaccines only target the spike protein (S), that provides the cellular entry of the virus through the ACE2 receptor (69, 70). However, this protein can accumulate a higher rate of mutations among other SARS-CoV-2 proteins, and thus may contribute to escape immunity (66, 71). To improve the protection against mutant strains and reduce the use of antibiotic, future COVID-19 vaccines should target other antigenic viral proteins with the capacity to induce less epitope variability (72, 73). For example, the effort to avoid a double health burden of “flu and COVID-19” has led to increased flu vaccination coverage and this may have influenced less antibiotic prescribing for flu-like infections and secondary bacterial complications.

## Impact of Other Measures for the Prevention of COVID-19 Transmission

**Figure 4:**
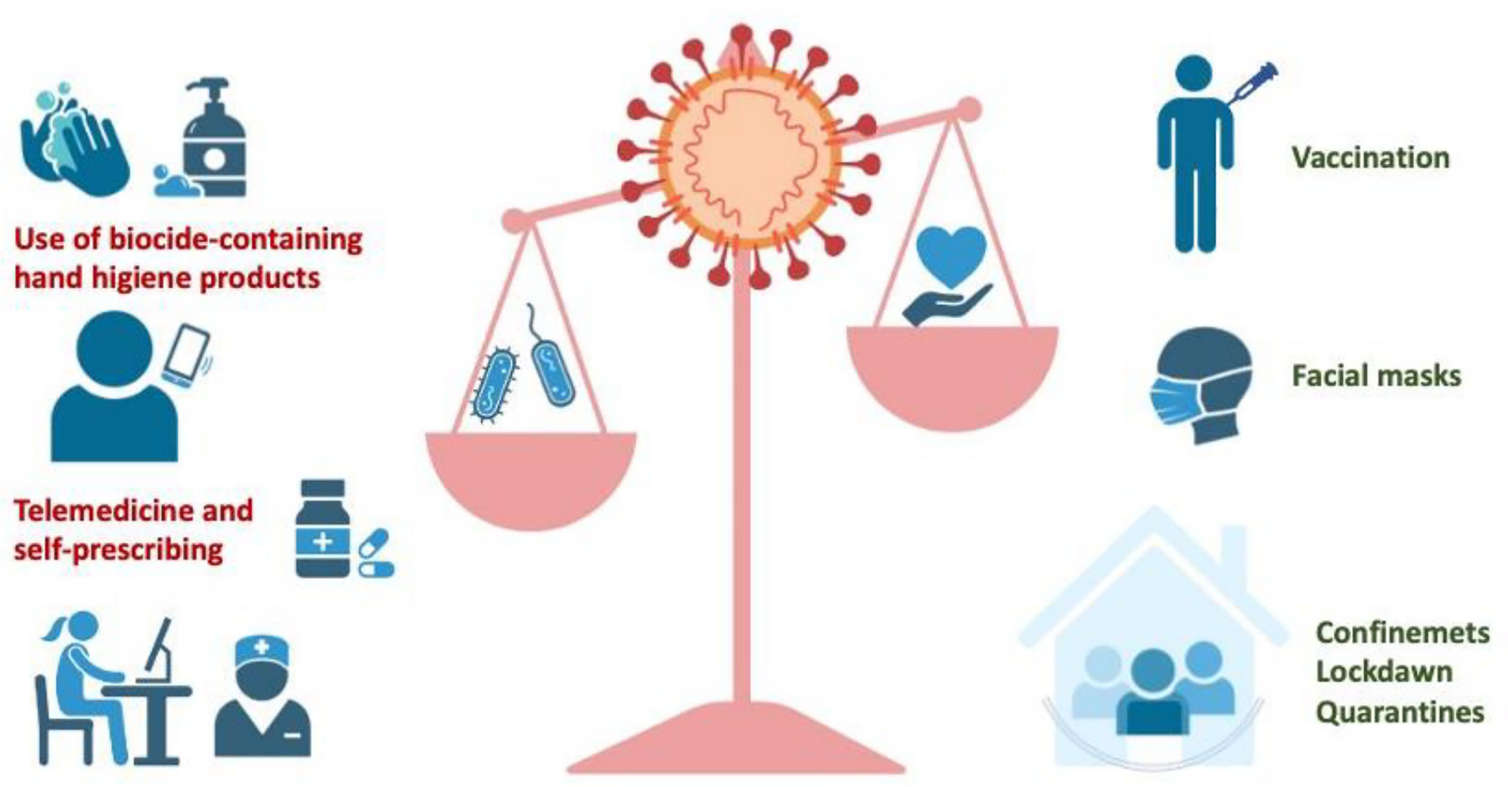
Measures that prevent the spread of SARS-CoV-2 infection may have an impact on the emergence of resistant microorganisms. Vaccination, the use of facial masks, and measures limiting social contacts may result in a decrease in antibiotic prescriptions, limiting the appearance of resistant microorganisms. However, the use of biocide-containing hand hygiene products, telemedicine, and self-prescribing may have increased the use of antibiotics and the release of biocides into the environment, facilitating the emergence of resistant microorganisms.

With the COVID-19 pandemic, measures such as lockdowns, physical distancing, travel restrictions, and quarantines implemented for persons in close contact with a positive have been implemented. These measures may have contributed to a reduction in the opportunities for transmission of many pathogens beyond SARS-CoV-2 (74). On the other hand, transmission within a local household or facility may be amplifying (75). Generally, the situation of confinement, and the limitation in leaving home, could have contributed to a decrease in the number of medical visits and, therefore, to a decrease in antibiotic prescriptions (76) limiting the appearance of resistance microorganisms (**Figure 4**).

In general, the use of facemasks is a key strategy for the effective prevention of airborne diseases. The use of facemasks potentially blocks airborne transmission routes and it is recognized as an effective containment measure in the COVID-19 pandemic: indeed, it prevents droplet dispersal when infected persons talk, sing, cough, or sneeze (77). Then, facemasks can reduce the risk of environmental contamination by respiratory droplets and the spread of the virus among people. In addition, the possible virus diffusion by contact can be avoided, due to the limitation of respiratory droplets that may deposit onto surfaces. Facemasks also provide a physical obstruction that prevents the wearer from touching his or her face, thereby reducing fomite transmission (78). In the pandemic scenario, mask use helps minimize the spread of SARS-CoV-2 infection by the wearer (79, 80). It is not surprising that health authorities recommend the use of facemasks to reduce the risk of environmental contamination and disease transmission. Finally, this reduces the need to use antimicrobials, and the risk of emerging resistant microorganisms (80).

With the pandemic, administrations and health authorities have promoted telemedicine, where physicians can prescribe medications. It is possible that this would force doctors to prescribe drugs without being able to auscultate, or take a sample, to establish a true diagnosis. In this scenario, it can be postulated that, on occasion, doctors may have over-prescribed antibiotics, as a conservative attitude (81–83). Although limiting social contacts, this measure could negatively impact the appearance of resistant microorganisms (**Figure 4**). The extended use of hand hygiene products containing biocides is another measure that could result in the release of chemicals into the environment facilitating the emergence of resistant microorganisms (**Figure 4**).

## The Need for New Antimicrobials

Most of the currently available antibiotics were discovered between 1940 and 1960 (84, 85). However, by the mid-1960s the rate of identification of new and efficient structures had declined dramatically (86). Until the 1980s, the lack of new discoveries of antimicrobials was compensated by the pharmacological study and optimization of existing antibiotics. Particularly the study of the biochemical mechanisms that describe both the action of drugs on their targets, and their associated resistance was largely investigated. This era was defined by the obtaining of a large number of optimized derivatives through the synthesis and chemical modification of existing antibiotics with improved activities and broader spectra (87). In the 1990s, a new wave of resistance has resulted in significant in projects to identify new classes of molecules in the antibiotic capacity (86). This discovery was based on the rational design of molecules, genomic analysis platforms, and combinatorial chemistry, coupled with computational tools. However, the race to generate new antibiotics by pharmaceutical companies was later abandoned. The main reason was the null permeability of the new compounds obtained, which prevented them from crossing the bacterial wall to exert their antibiotic activities (88). During evolution, the molecular tools necessary to develop a resistance mechanism have always been accompanied by the natural ability of microorganisms to generate antibiotics. Thus, we could predict that eventually, new resistance mechanisms will emerge in response to the use of the latest generation of antibiotics (89). A major concern for microbiologists and infectious disease authorities is the continuous increase in resistance and the rapid spread among strains of a microorganism and the lack of effective antibiotics (90).

The development and manufacture of new antimicrobials is a long process. It can take more than 10 years from discovery to commercialization of a new antibiotic (91, 92). This, coupled with the fact that bacteria can develop resistance mechanisms that make them unusable, makes them unattractive to the pharmaceutical industry. Furthermore, this low profitability would be influenced by the possibility of marketing generic antibiotics ten years after their introduction on the market (93, 94).

Due to the current difficulties in obtaining new classes of antibiotics, we are now approaching a situation of inability to control infections (95). If new drugs are not developed, deaths due to antibiotic resistance are expected to exceed 10 million per year by 2050 (96). The problem is that infectious diseases are estimated to become the leading cause of death, ahead of cancer and cardiovascular diseases (89).

In the current COVID-19 pandemic, the focus of the pharmaceutical industry has been to develop vaccines and effective antimicrobials against SARS-CoV-2 (97). However, we should not forget the need for new antimicrobials, as the emergence of AMR is likely to increase, impacting COVID-19 morbidity and mortality. Therefore, any antimicrobial strategy to find new structures of more efficient anti-infectives should be promoted. Compounds with a broad spectrum of action and less resistance are particularly desirable.

## Antimicrobial Peptides and Antibodies as Anti-Infective Drugs

Anti-infective treatment is based on the principle of targeting molecular pathways that cause infection but do not impair bacterial growth. In the face of a shortage of new antibiotics, antimicrobial peptides and specific antibodies, are gaining attention as Nature-derived anti-infective agents with great clinical potential (98, 99). Antimicrobial peptides (AMPs) and antibodies both play an important role in the defense against foreign microorganisms and are part of innate and acquired immunity, respectively. They both have less capacity to generate resistance in target microorganisms and can be administered together with other antibiotics or antimicrobial compounds. AMPs are a rapid, non-specific means of combating a wide variety of bacteria, fungi, viruses, and even protozoa (100). Specific antibodies are essential macromolecules for the adaptive immune systems of all vertebrates. Defense blood cells are also important producers of AMPs, where they constitute part of the non-oxidative effector mechanisms against potential pathogens (101). AMPs are synthesized mainly in epithelial tissues regularly exposed to microbial attacks such as skin, intestine, and lungs. In the body, AMPs are synthesized up to a hundred times faster than antibodies and at much lower metabolic cost, can be stored in high concentrations, are available for immediate action, and are released or produced when cells are stimulated by contact with microorganisms (102). However, antibodies only recognize a single infectious agent, and even while mutations of that agent may render the antibody useless, this does not affect other similar agents and does not contribute to the spread of resistance (103). Faced with a market where cheap and effective antibiotics are accessible, the manufacture of AMPS and antibodies for biomedical applications exhibit inherent constraints; such as high production cost, storage conditions, and dosage for administration (104) (**Table 1**).

**Table 1:** Different features of antibiotics, antibodies, and antimicrobial peptides as anti-infective agents for infectious diseases.

Feature	Antibiotics	AMP	Antibodies
Cost of production	Low	High	High/moderate
Storage conditions	Room temperature	Refrigerated	Refrigerated
Administration	Orally (tablets)	Topical/systemically	iv/sc injection
Target specificity	Narrow	Broad/narrow	Narrow
Resistance	Yes	Difficult	No
Structure	Simple, homogeneous,well-characterized	Diverse, homogeneous, well-characterized	Complex, heterogeneous, and less characterized
Molecular weight	Low (<1kDa)	<5 kDa	High (>50 kDa)
Degree of instability	Low	High	Low
Risk of immunogenicity	Low	Low	High
Mechanisms of action	Generally specific	Variable or still unknown	Specific

**Figure 5:**
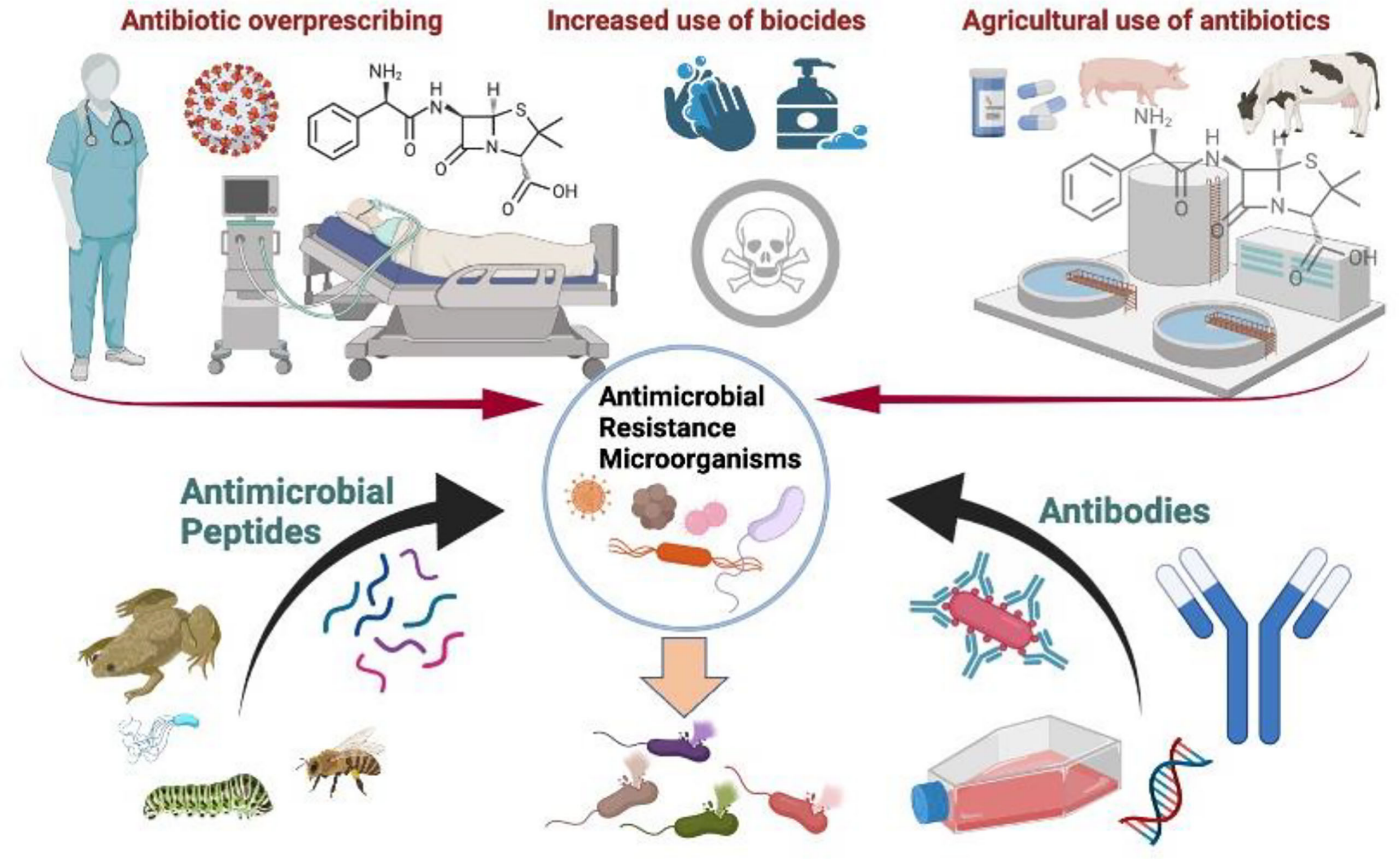
In the current COVID-19 scenario, antibody-based treatments such as pathogen-specific monoclonal antibodies (MAbs) and antimicrobial peptides (AMPs) could limit the development of resistant microorganisms as they are considered to generate little or no resistance.

In the context of COVID-19, excessive antibiotic prescribing, the increased use of biocides, and the agricultural use of antibiotics can contribute to the emergence of resistant microorganisms. Conventional antibiotics generally target metabolic enzymes that may selectively develop resistance, whereas AMPs and antibodies kill microbes or neutralize infectious pathogens, thus making it inherently more difficult for the organisms to develop resistance. They can be regarded as safe and effective templates for the generation of future antimicrobials (104) (**Figure 5**).

### Antibodies and Antibody-Derived Therapies

Antibodies and antibody-derived therapies provide an intriguing set of tools and properties for killing or neutralizing infectious pathogens, lysing infected cells, or modulating the immune system to allow effector cells to escape immunosuppressive events and contribute to infection removal (99). The flexibility to generate antibodies against any target, the ability to alter effector functions, half-life, and the size of treatment units, are particularly well suited for customizing therapies to specific infectious agents (103). Proteins on microbe surfaces, toxins, and other virulence factors can all be targets for antibodies. Enzyme areas crucial for microbial metabolism can also be targeted by antibody design, being able to influence the activity of antigen molecules without necessarily affecting the immune response (105).

Antibody-based treatments, such as pathogen-specific monoclonal antibodies (MAbs), have demonstrated promise in the treatment of bacteria (e.g., MRSA) and viruses that are resistant to conventional antibiotics (106). Over 40 antibody-based drugs have been authorized or are pending approval. The selectivity of the antibodies translates into the very minimal off-target binding and hence very few side effects, including the gastrointestinal problems sometimes associated with antibiotics due to their wide impact on the gut bacterial flora in the environment (99). However, certain characteristics of monoclonal antibodies make them less suited when compared to other antimicrobial therapies (103). The first is the high production cost. Because of the widespread use of broad-spectrum antibiotics, antibody-based treatments are less interesting to the pharmaceutical industry. Antibodies are proteins, they must be handled with care, kept cold, and be injected intravenously or subcutaneously (103). Antibiotics, on the other hand, are generally formulated as pills or tablets that may be used orally and stored at room temperature. Antibiotics target general mechanisms in bacteria, such as cell wall formation, and can be effective against a wide range of bacteria. Antibodies, on the other hand, are very specific to a single virus, bacterium, or bacterial subtype. Antibody therapy provides an immediate level of immunity, and a clear diagnosis must be made before starting treatment with a monoclonal antibody. For example, antibody treatment has been administered to patients with recurrent *C. difficile* infections (107). However, several advancements, including dosage, cost, and biologic stability, remain for the normal use of MAb administration for AMR prevention (103). A larger and more realistic context should be used to evaluate the trade-off between antibody treatments and antibiotics in terms of cost and convenience of use in the clinic (103). This includes the current COVID-19 scenario surrounding the development of resistance to antimicrobial drugs (97). A few MAb-based drugs are currently authorized for use by the US FDA in infectious disorders (**Table 2**). New antibody-based therapies to treat and prevent bacterial-associated pneumonia are being developed (112). Chicken polyclonal antibodies against SARS-CoV-2 have been proposed as anti-infective treatments for COVID-19 (113). Antibody-based drugs will continue to play an essential role in the development of new infectious disease treatments in the future (99). It is envisaged that some of these initiatives will show clinical success and hence provide the foundation and enthusiasm for this process (114).

**Table 2:** MAbs approved by the US FDA for treatment of infectious diseases.

mAb	Target	Format	Indication	Ref
Palivizumab	RSV	Humanized IgG1	Prevention of respiratory syncytial virus infection	(108)
Raxibacumab	*B. anthrasis* PA	Human IgG1	Anthrax infection	(109)
Bezlotoxumab	*C. difficile* enterotoxin B	Human IgG1	Prevention of *Clostridium difficile* infection recurrence	(107)
Obiltoxaximab	*B. anthrasis* PA	Chimeric IgG1	Prevention of inhalational anthrax	(110)
Ibalizumab	CD4	Humanized IgG4	HIV infection	(111)

### Antimicrobial Peptides

AMPs usually exhibit broad-spectrum antimicrobial activity, although some AMPs may exhibit bacteriostatic, immunomodulatory, anti-inflammatory, and antitumor activity (115, 116). All these properties make AMP substances with great pharmacological potential (117, 118). One advantage of AMPs is their action on biological targets, other than traditional antibiotics, and their multiple mechanisms of action (117). Although AMPs are very diverse structurally; many show some common features, such as their positive net charge and their high hydrophobicity. These two properties allow them to interact with lipid membranes (119). The net positive charge is essential to interact electrostatically with the negatively charged environment of bacterial membranes and to stabilize the binding. On the other hand, hydrophobicity makes these peptides more permeable to the lipid bilayer and they can bind to each other and form pores in bacterial membranes (102). The formation of pores in the bacterial membrane leads to its destabilization and allows the entry and exit of metabolites, inducing bacterial lysis. Many antimicrobial peptides act on the bacterial membrane that has evolved. To generate resistance to AMPs, bacteria must restructure the architecture of their cell membranes, a process that will take several generations and multiple mutations. This fact means that AMPS are considered to generate little or no resistance (102, 119).

Structurally, antimicrobial peptides are widely categorized into four large classes based on the polypeptide chain bonding types: class O (circular), class P (resembling a P-shape, where a chemical bond is formed between the sidechain of one amino acid and the backbone of another amino acid in the chain), class S (containing a chemical bond between different sidechains), and class L (linear peptides). This classification is also named UCBB, UCSB, UCSS, and UCLL, respectively (120). Some antimicrobial peptides have a simple helix or sheet structure, whereas others are more complex. According to how cells can synthesize AMPs, they can be classified as ribosomal or non-ribosomal. Non-ribosomal peptides are generally synthesized by bacteria and assembled by cytosolic multi-modular enzymes, whereas ribosomal peptides are gene-encoded and usually result from the cleavage of a pre-propeptide (102).

The large diversity of cationic peptides has been hypothesized to derive from their antibacterial role in combating the distinct pathogenic microorganism issues faced by each host organism (119). Nature contains an almost unlimited number of peptide drugs that need to be pharmacologically characterized (121, 122). Templates for the development of new antimicrobial agents can be found from creatures that live in germ-filled habitats, which can penetrate bacterial membranes (123, 124). The Antimicrobial Peptide Database APD (https://aps.unmc.edu/home) collects the characteristics and activities of more than 3000 antimicrobial peptides from six life kingdoms. Animal-derived AMPs such as magainins, dermaseptins, and other AMPs isolated from frog skin, are being studied as potential therapies for skin infections as both therapeutic and preventative agents in humans. Because topical antimicrobial peptide treatment prevents systemic toxicity, many antimicrobial peptides have been developed as topical applications (102). Despite the growing interest in AMPs as broad-spectrum, non-resistance-generating antibiotics, only a few AMPs have been approved by the US FDA for clinical use (117, 125) (**Table 3**).

**Table 3:** Natural AMPs approved by the US FDA for clinical use.

AMP	Source	Mechanism	Target	Indication	Ref
Nisin	*Lactococcus lactis*	Inhibit cell wall synthesis	Broad spectrum activity	Treatment of stomach ulcer and colon infections	(126)
Melitin	*Apis mellifera*	Membrane disruption	Broad spectrum activity	Relieving pain and swelling	(127)
Gramicidin	*Brevibacillus brevis*	Membrane disruption	Gram-positive bacteria	Ophthalmic purposes	(128)
Daptomycin	*Streptomyces roseosporus*	Inhibits cell wall synthesis	Gram-positive bacteria	Skin infections	(129)
Polymyxins	*Paenibacillus polymyxa*	Insertion of the AMPs into the membrane	MDR Gram-negative bacteria	Eye and wound infections	(130)

Nisin has been approved for the treatment of stomach ulcer and colon infections. Currently, nisin is the only non-ribosomal antimicrobial peptide commonly utilized in food preservation being an additive for the agricultural and food industries. Daptomycin is a 13 amino acid cyclic lipopeptide generated by the bacteria *Streptomyces roseosporus*. It has bactericidal effect against Gram-positive bacteria, including those that are resistant to antibiotics. The combination of peptide antibacterial capabilities with antibiotics has the potential to reduce the development of resistance. For example, when the antimicrobial peptide. AMPs may interact synergistically with immune system components in addition to having synergistic activity when coupled with antibiotics and could be potential alternatives to conventional antibiotics due to their favorable safety profile and low or limited ability to induce bacterial resistance (102). Cathelicidins are one of the most promising antimicrobial peptides. However, there are intrinsic characteristics of antimicrobial peptides that still make them unattractive to the pharmaceutical industry: such as their stability, adsorption, inability to cross epithelial and skin barriers, potential immunogenicity, and the difficulty of conferring oral bioavailability to these molecules (114). Many antimicrobial peptides are nephrotoxic due to their high therapeutic dosage and several AMPs have failed in Phase III clinical trials due to lack of clear efficacy or lack of superiority over conventional treatments (117).

## Conclusions

Public actions to mitigate COVID-19 have resulted in a change in the public’s behavior regarding the adoption of preventive measures. Some measures, such as face masks, social distancing, and increased hand hygiene will likely diminish AMR. In contrast, over-prescription of antibiotics, nosocomial infections, and telemedicine could have contributed to an increase in AMR (131–133). Everything seems to indicate that this new virus, SARS-CoV-2, is contributing to worsening the current situation concerning the emergence of resistant microorganisms (19, 21). AMR demands a “one health” approach, which recognizes that human and animal health are interrelated, and that infections are transmitted from people to animals and vice versa. These changes in the everyday use of antibiotics and other substances could result in the release of antibiotics and resistant bacteria into the ecosystem *via* contaminated water, food, or excretion. Consequently, the spread of AMR across many domains of one health, such as healthcare, agriculture, and the environment, might be affected. A global vision of the pandemic impact on AMR is not known yet and can only be speculated at this point. However, it is reasonable to suppose that new treatments and vaccines designed to restrict the spread of SARS-CoV-2 should limit the development of resistant microorganisms and vice versa. The decline in the investment and lack of innovation of antibiotics also favors the emergence of antimicrobial-resistant organisms (89, 133). Current vaccines against COVID-19 have not been implemented globally. There are still countries where there is a very little vaccination (97, 134). The lack of sterilizing vaccines to prevent human-to-human transmission might increase the risk of the emergence of new variants of SARS-CoV-2 and enhance the adverse impact of COVID-19 on AMR (62). In the context of anti-infective therapy, Nature derived anti-infectives have the potential to be helpful as a part of the arsenal to combat AMR infections (114). They are particularly useful as can be co-delivered with existing antibiotics. More research and development are still required to view the true potential of these agents as powerful tools against drug-resistant pathogens. However, new antibody-based drugs or AMPs might establish their relevance and make a difference in some infectious disease sectors. If these expectations are satisfied, a new generation of antimicrobial medications that are flexible, powerful, and long-lasting will be accessible soon.

## Author Contributions

All the authors of this manuscript have substantially contributed to the concept, literature mining, writing and methodology of the review, provided critical feedback and revised the manuscript critically. All authors contributed to the writing or revision of the final manuscript. **JMPL**: Conceptualization, literature survey, writing-original draft preparation, prepared the tables and figures, data validation, revised the manuscript, formal analysis, supervised the drafting process of the review, project administration and funding acquisition. **UA**: Conceptualization/conceived the study idea, planned and designed the review structure, wrote the first draft of the manuscript, visualization, response, suggestions, revision, final draft. **SG-A**: Writing—review & editing, prepared tables and figures, completed the critical revision of the entire manuscript. **MRL**: Writing-review & editing, completed the critical revision of the manuscript, response, suggestions, and arranged references. **AD**, and **EB**: Writing-review & editing, overall proofreading, response, valuable suggestions. **ANN**: Writing-review & editing, response, valuable suggestions. All authors have read and approved the final version of the manuscript for submission to this journal.

## Funding

This research was funded by projects “Agencia Canaria de Investigación, Innovación y Sociedad de la Información (ACIISI) del Gobierno de Canarias” (project ProID2020010134), and CajaCanarias (project 2019SP43). MRL acknowledges contract (IMMUNOWINE) financed by Cabildo de Tenerife, Program TF INNOVA 2016-22 (with MEDI & FDCAN Funds). AMN is a recipient of a postdoctoral Marie Curie fellowship under grant agreement 101030604 (IGYMERA). The funders had no role in the design of the study; in the collection, analyses, or interpretation of data; in the writing of the manuscript, or in the decision to publish the results.

## Conflict of Interest

The authors declare that the research was conducted in the absence of any commercial or financial relationships that could be construed as a potential conflict of interest.

## Publisher’s Note

All claims expressed in this article are solely those of the authors and do not necessarily represent those of their affiliated organizations, or those of the publisher, the editors and the reviewers. Any product that may be evaluated in this article, or claim that may be made by its manufacturer, is not guaranteed or endorsed by the publisher.
