# Corrigendum: Antimicrobial resistance in the COVID-19 landscape: is there an opportunity for anti-infective antibodies and antimicrobial peptides?

**DOI:** 10.3389/fimmu.2024.1365827

**Published:** 2024-01-26

**Authors:** José M. Pérez de la Lastra, Uttpal Anand, Sergio González-Acosta, Manuel R. López, Abhijit Dey, Elza Bontempi, Antonio Morales delaNuez

**Affiliations:** ^1^ Biotechnology of Macromolecules, Instituto de Productos Naturales y Agrobiología, IPNA (CSIC), San Cristóbal de la Laguna, Spain; ^2^ CytoGene Research & Development LLP, Barabanki, Uttar Pradesh, India; ^3^ Department of Life Sciences, Presidency University, Kolkata, India; ^4^ National Interuniversity Consortium of Materials Science and Technology (INSTM) and Chemistry for Technologies Laboratory, Department of Mechanical and Industrial Engineering, University of Brescia, Brescia, Italy

**Keywords:** SARS-CoV-2, antibiotic resistance, one health approach, global health, antibiotic discovery, antimicrobial peptides, environmental contamination, vaccination

In the published article, there was an error in the affiliation information about Uttpal Anand [2]. Instead of “Department of Life Sciences, Ben-Gurion University of the Negev, Beer-Sheva, Israel”, it should be “**CytoGene Research & Development LLP, Uttar Pradesh, India**”.

The authors apologize for this error and state that this does not change the scientific conclusions of the article in any way. The original article has been updated.

